# Neonatal lamb mortality: major risk factors and the potential ameliorative role of melatonin

**DOI:** 10.1186/s40104-020-00510-w

**Published:** 2020-11-05

**Authors:** Tom Flinn, David O. Kleemann, Alyce M. Swinbourne, Jennifer M. Kelly, Alice C. Weaver, Simon K. Walker, Kathryn L. Gatford, Karen L. Kind, William H. E. J. van Wettere

**Affiliations:** 1 Davies Livestock Research Centre, School of Animal and Veterinary Sciences, The University of Adelaide, Roseworthy, SA Australia; 2 Turretfield Research Centre, South Australian Research and Development Institute, Rosedale, SA Australia; 3 Robinson Research Institute, Adelaide Medical School, The University of Adelaide, Adelaide, SA Australia

**Keywords:** Lamb survival, Melatonin, Neonatal mortality, Reproduction, Sheep

## Abstract

High incidences of pre-weaning mortality continue to limit global sheep production, constituting a major economic and welfare concern. Despite significant advances in genetics, nutrition, and management, the proportion of lamb deaths has remained stable at 15–20% over the past four decades. There is mounting evidence that melatonin can improve outcomes in compromised ovine pregnancies via enhanced uterine bloodflow and neonatal neuroprotection. This review provides an overview of the major risk factors and underlying mechanisms involved in perinatal lamb mortality and discusses the potential of melatonin treatment as a remedial strategy. Supplementing pregnant ewes with melatonin enhances uterine bloodflow and fetal oxygenation, and potentially birthweight and neonatal thermogenic capacity. Melatonin freely crosses the ovine placenta and blood-brain barrier and provides neuroprotection to the fetal lamb during periods of chronic and acute hypoxia throughout gestation, with improved behavioural outcomes in hypoxic neonates. The current literature provides strong evidence that maternal melatonin treatment improves outcomes for lambs which experience compromised in utero development or prolonged parturition, though to date this has not been investigated in livestock production systems. As such there is a clear basis for continued research into the effects of maternal melatonin supplementation during gestation on pre-weaning survival under extensive production conditions.

## Introduction

High pre-weaning mortality limits sheep production globally, with the proportion of lamb deaths across many countries and systems remaining stable at 15–20% over the past 40 years [1]. In Australian flocks, average losses of 10% and 30% for singleton and twin lambs, respectively [2, 3], cost the industry an estimated $540 million annually in lost production and in amelioration strategies [4]. Predominant causes of death can differ between regions depending on exposure to risk factors such as disease or extreme weather [1], though there is a consensus that the majority of losses occur in the first 3 d post-partum [2, 5, 6]. In extensively grazed flocks, around half of all losses are parturition-related, comprising stillbirth (21%), birth injury (18%), and dystocia (9%), followed by starvation-mismothering (25%), death in utero/prematurity (10%), predation (7%), and cold exposure (5%) [6]. Lamb birthweight and litter size, along with dam breed, are the main risk factors for neonatal loss [3]. The relationship between birthweight and survival is curvilinear, with more deaths occurring in lambs born with weights outside the ideal range of 4.0–6.0 kg [3, 7], though predominant causes differ between underweight and overweight lambs. Heavy lambs, especially singletons, have higher rates of dystocia and stillbirth due mainly to feto-pelvic disproportion [6]. Conversely, low birthweight lambs, especially from multiple pregnancies, exhibit higher rates of birth injury, starvation, and hypothermia [6, 8, 9]. This is partially attributable to lighter lambs being slower to stand and suckle after birth [10], and less able to maintain homeothermy [11]. Nevertheless, greater losses among twins cannot be attributed to birthweight alone, as these losses are consistently higher than found in singletons even at the same weight [7]. This reflects the impact of prolonged birth and intrapartum asphyxia due to a weak dam and/or fetal entanglement or malpresentation, various ewe-lamb behavioural interaction factors, and the limited capacity of the ewe to meet the nutritional requirements of both twins before and after birth [12]. This review focuses on causes of hypoxia and low birthweight, their impacts on the neonatal lamb, and discusses the potential of melatonin treatment as a remedial strategy.

### Ease of parturition, intra-partum asphyxia, and oxidative stress

Stillbirth and birth injury are largely the consequence of intrapartum asphyxia, the probability of which increases with duration of parturition and can be 16-fold higher for twin lambs vs. singletons [8]. Primarily, this manifests as hypoxic-ischaemic encephalopathy (HIE); a biphasic condition characterised by impaired cerebral oxygenation and widespread neurological damage [13, 14]. During the initial phase (ischaemia), reduced cerebral blood flow leads to decreased cellular oxygen availability, glucose, and adenosine triphosphate (ATP) levels, triggering an increase in anaerobic glycolysis and subsequent lactate production, cellular acidification, and ion pump failure. The subsequent neuronal depolarisation, resulting from failure of Na^+^ and K^+^ pumps, triggers the release of glutamate and an additional intracellular influx of Na^+^ and Ca^2+^, the overload of which induces mitochondrial injury, disrupted protein synthesis, DNA fragmentation, cerebral oedema, and ultimately necrotic or apoptotic cell death [14, 15]. Cellular damage during acute ischaemia occurs primarily in the brain, due to its high rate of oxygen consumption, and the severity of damage is proportional to the duration of ischaemia. If ischaemia is prolonged, cellular damage often extends to the myocardium, renal tubules, and liver tissues [13, 16].

Following ischaemia (primary energy failure) there is a latent period before the initiation of reperfusion injury (secondary energy failure). Secondary energy failure can occur anywhere from 6 to 48 h after primary energy failure depending on severity of the initial hypoxic insult; whereby increased severity shortens the latent period between phases. Secondary energy failure is induced, paradoxically, by re-establishing cellular oxygen delivery [14, 15]. Restoration of blood flow to ischaemic tissues, although essential for cellular survival, induces production of reaction oxygen species (ROS) by mitochondrial enzymes, particularly superoxide (O_2_•^−^) and hydrogen peroxide (H_2_O_2_), the latter of which is converted to the highly reactive hydroxyl radical (•OH) via ferrous iron [17]. Restoration of blood flow also delivers platelets and leukocytes to the cell which, when activated, release additional ROS and hydrolytic enzymes [15]. High levels of ROS inflict significant cellular damage via peroxidation of organelle and cell membrane lipids, and the oxidation of proteins, polysaccharides, and DNA, leading to fragmentation, base modifications, or strand breaks, and ultimately to apoptotic cell death [17]. This damage is characterised by meningeal haemorrhaging, central nervous system lesions, and impaired neuro-motor activity of the neonate [13, 18, 19]. Severe intrapartum asphyxia, if not fatal, can cause long-term motor and neurodevelopmental disabilities [20], as well as impaired organ function via damage to heart, kidney, and liver tissues [13, 16].

Hypoxic brain injury following prolonged parturition has significant negative impacts on the neonatal lamb’s behaviour and metabolism. These include increased latency to stand and suckle, reduced levels of thyroid hormones and hence impaired thermogenesis, reduced thermoregulatory capacity, and higher mortality in the first week after birth compared with lambs from short, uncomplicated births [8, 21]. Additionally, neurological impairment may negatively affect frequency of neonatal vocalisation and the maternal response, thereby increasing the likelihood of maternal rejection and subsequent starvation [21–23]. Further, ewes that experience difficult or prolonged parturition are slower to display maternal bonding behaviours and are more likely to reject their lambs [10, 21]. Dystocic ewes are also susceptible to pelvic obturator nerve damage via pressure from lambs and/or human hands during obstetric intervention, leading to hind limb paralysis that can persist for several days and prevent ewes standing after birth to mother their lambs [24]. Prolonged parturition therefore impairs early suckling activity and colostrum ingestion by the lamb.

### Neonatal lamb thermoregulation and vitality

The abrupt change in the lamb’s ambient temperature at birth requires as much as a 15-fold compensatory increase in endogenous thermogenesis [25], especially in cold environments where heat loss is exacerbated by wind velocity, humidity, and evaporation of amniotic fluid from the birth coat [7, 26]. The activity of peri-renal and peri-cardial brown adipose tissue (BAT) constitutes the lamb’s primary heat source at birth, providing over 50% of total bodily thermogenesis, with the remainder from muscle contraction via shivering and locomotion [27, 28]. Heat is generated rapidly within BAT cells via uncoupling of the electron transport chain from ATP synthesis; a process mediated by neural and endocrine networks which activate mitochondrial uncoupling protein 1 (UCP1) in response to cold exposure or acute feed intake [29]. Unlike liver and muscle glycogen reserves, which comprise around 9.6 g/kg bodyweight at term irrespective of absolute birthweight, lipid reserves are highly variable, such that an ‘underweight’ (2.90 kg) lamb has disproportionately lower levels of available lipid compared with a ‘normal’ (4.25 kg) lamb at term (4 vs. 12 g/kg, respectively) [30]. Lighter lambs are also more susceptible to rapid heat loss due to their higher surface area: bodyweight ratio [11, 25, 26]. This association between birthweight, thermoregulatory capacity, and lamb survival to weaning has been described in several studies [11, 31, 32]. Further, low rectal temperature at birth is associated with failure to stand and seek the udder [33]. Standing quickly after birth is important to reduce convective heat loss from the wet lamb to the ground, and suckling bouts also raise core body temperature [34], with failure to suck leading to death by starvation or secondary hypothermia [35]. Clearly, much of the lamb’s chance of survival is determined before birth, as they require sufficient birthweight and accumulated BAT and glycogen reserves to maintain homeothermy while transitioning from these endogenous energy sources to energy from colostrum and milk.

### Fetal development phases and risks

Fetal development is characterised by three major growth phases: embryonic, placental, and fetal. In sheep, the embryonic phase spans mating until 30 d post-conception (gestational day 30, gD30), during which embryonic attachment occurs at gD16–21 [36, 37]. Placental development begins around gD30 when the chorion fuses to endometrial caruncles to form individual placentomes, the number of which is fixed at this stage and remains unchanged through the remainder of gestation, although placentome weight continues to increase until around gD90 [38]. Following formation of placentomes, the vascular density in maternal caruncles increases markedly from gD40 until mid-gestation, before slowing through late gestation, with small increases in capillary number and a 2- to 3-fold increase in capillary diameter occurring between gD50 and gD140. Conversely, the vascular density of fetal cotyledons remains constant until mid-gestation, and increases significantly thereafter, with a 12-fold increase in capillary number and 6-fold increase in total capillary area occurring between gD50 to gD140 [39]. These changes are concurrent with increases in fetal growth rate from gD100 until term (gD145–150), which requires an equivalent increase in maternal energy intake to maintain adequate delivery of oxygen and nutrients to the fetus [36, 40]. In normal ovine pregnancies there is a threefold increase in uterine bloodflow (from 0.4 to 1.2 L/min) during the latter half of gestation [41]. Chronic abnormalities in uterine blood flow or placental vascularity lead to placental insufficiency, intrauterine growth restriction (IUGR), and reduced birthweight [42], and the associated neonatal risk factors outlined previously.

Impacts of compromised fetal blood supply extend beyond reduced birthweight alone. Growth restriction is also associated with reductions in total brain cell count, myelination, and brain and cortical grey matter volume. These reductions can influence neonatal behaviour via motor and sensory impairment, thus increasing the risk of perinatal death [20, 43, 44]. Even brief (acute) periods of fetal hypoxia have negative impacts on the fetal brain, namely increased •OH release in cortical grey matter, cerebral white matter damage, and death of neuronal populations in the cerebellum, hippocampus, and cortex, via the oxidative stress mechanisms described previously [14, 15, 17, 20, 45]. As well as growth restriction and neurological impairment, compromised placental nutrient supply can negatively impact thermoregulatory capacity of the neonatal lamb. The mid-late gestation period is critical for development of perirenal and pericardial BAT reserves, which grow rapidly from gD70 until gD110–120 [27] and increase in protein content from gD120 until term [46]. Consequently, maternal undernutrition, restricted placental size, or impaired uterine bloodflow during this period reduces the weight of BAT depots and whole body lipid near term, independent of effects on fetal weight [27]. Maternal nutrient restriction (50%) from gD115 to term reduces lamb perirenal BAT weight relative to body weight by 15%, with a 2.5-fold reduction in UCP1 expression [47]. Longer-term restriction throughout the whole of pregnancy, induced by surgical removal of endometrial caruncles before mating and consequent placental restriction, induces similar reductions of 36–37% in both birthweight and BAT weight [48]. These findings together with the previous sections indicate that an intervention that improves fetal growth and tolerance of hypoxia is likely to improve neonatal survival of lambs, particularly in restricted pregnancies such as in twin-bearing ewes.

### Melatonin

Melatonin (N-acetyl-5-methoxytriptamine) is an endogenous hormone which plays a pivotal role in mediating diurnal and seasonal patterns of animal physiology and behaviour [49]. The release of melatonin from the pineal gland is regulated by the suprachiasmatic nucleus of the hypothalamus in response to signalling from the retina, whereby secretion occurs exclusively at night and is inhibited during the day. Seasonal changes in photoperiod alter the duration of daily melatonin release, which in turn triggers seasonal changes in behaviour [49]. Effects of melatonin on various tissues are mediated by G-protein-coupled receptors MT1 and MT2, which induce varying responses depending on tissue type [50]. As well as regulation of circadian and seasonal rhythms, melatonin is a potent antioxidant, acting through several pathways. Firstly, melatonin directly scavenges a wide range of ROS and neutralises reactive nitrogen species, and the metabolites produced from this process then also act as ROS scavengers [51]. Additionally, melatonin and its metabolites upregulate antioxidant enzymes including glutathione peroxidase, glutathione reductase, and superoxide dismutase, and downregulate pro-oxidant enzymes including lipoxygenases [17]. Further, melatonin reduces ROS formation by increasing the efficiency of electron movement between mitochondrial respiratory complexes [51]. These potent antioxidant properties formed the rationale for recent animal studies, including several in pregnant or neonatal sheep. Although most of these studies were designed to assess the potential for melatonin as a treatment for human infants [52], they offer compelling evidence to warrant further investigation into the use of melatonin for improving ovine reproductive performance, particularly regarding the potential to reduce lamb mortality.

#### Neonatal melatonin reduces consequences of chronic and acute hypoxia in lambs

Several studies have shown benefits of treating the neonatal lamb with melatonin after chronic hypoxia during compromised pregnancies as well as after acute hypoxia mimicking birth asphyxia (Table 1). Pregnancies maintained under chronic hypobaric hypoxia lead to restricted fetal growth and high rates of pulmonary hypertension and endothelial dysfunction in the neonate. In studies conducted at high altitude (3600 m), feeding melatonin (1 mg/kg daily) to neonatal lambs conferred a range of benefits by improving vascular function. Outcomes include improved carotid artery bloodflow and cerebral perfusion [53], increased vascular density and luminal surface area of pulmonary arteries, and reduced pathological vascular remodelling in response to oxygenation changes [54]. Similarly, melatonin decreased pulmonary arterial pressure and contractile response to vasoconstrictors, and increased endothelium-dependent and muscle-dependent pulmonary vasodilation [55]. These outcomes were accompanied by a reduction in oxidative stress markers via upregulation of antioxidant enzymes and diminishing pro-oxidant sources [53–55]. Neonatal melatonin treatment (60 mg, intravenous or transdermal patch) of lambs born following acute perinatal asphyxia via umbilical cord occlusion (UCO) significantly reduced HIE symptoms when treated lambs had lower rates of apoptotic cell death in white and grey matter, lipid peroxidation, and neuroinflammation in brains measured 72 h post-partum. This was reflected in marked behavioural improvement compared with untreated asphyxiated lambs, specifically reduced latency to stand and suckle, a higher proportion of lambs suckling successfully, and fewer seizures [56]. While these studies support a potential role for melatonin in improving outcomes for compromised neonates, the highest risk period for oxidative damage to the fetus/lamb in extensive production systems is during gestation and parturition. However, the continuous monitoring required for timely identification and treatment of hypoxic neonates is unrealistic in this setting. As such, for an intervention to be practical under commercial conditions, it needs to be provided to the fetus during late gestation to provide protection through periods of chronic hypoxia as well as acute hypoxia at parturition. Importantly, melatonin diffuses freely across the placenta and blood-brain barrier [57, 58], as evidenced by fetal circadian rhythms, whereby melatonin levels are similar to that of the host ewe but at lower amplitude and slightly delayed [58]. Therefore, maternal supplementation offers a clear access route for prenatal delivery of melatonin to the developing fetus.

**Table 1:** Experimental studies of neonatal melatonin supplementation in ovine models of prenatal or neonatal hypoxia

Publication	Methodology	Melatonin dose rate/timing	Lamb outcomes
Herrera et al. [53]	• Ewes subjected to chronic hypobaric hypoxia (altitude 3600 m) from pre-conception until end experiment • Graded oxygenation protocol to assess acute neonatal responses to oxygen concentration (controlled gas levels in polyethylene bag over lamb’s head) after 7 d melatonin treatment • Tissue collected for analysis 12 d post-partum	• Oral to lamb: 1 mg/kg daily at 18:00 h from d 4–12 post-partum • Treatments: - Control (EtOH vehicle) - MEL	In MEL lambs compared with controls: • Greater fractional growth during first 3 d of treatment • Greater carotid bloodflow at all PO_2_ levels • Improved vascular responses to potassium, serotonin, methacholine, and melatonin itself • Improved endothelial response in isolated middle cerebral arteries via NO-independent mechanisms • Lower oxidative stress (nitrotyrosine) level in middle cerebral arteries
Astorga et al. [54]	• Ewes subjected to chronic hypobaric hypoxia (altitude 3600 m) from pre-conception until end experiment • Graded oxygenation protocol (controlled gas levels in polyethylene bag over lamb’s head) to assess acute neonatal responses to oxygen concentration after 7 d melatonin treatment • Tissue collected for analysis 12 d post-partum	• Oral to lamb: 1 mg/kg daily at 20:00 h from d 3–12 post-partum • Treatments: - Control (EtOH vehicle) - MEL	In MEL lambs compared with controls: • Reduced pulmonary pressor response to graded oxygenation changes • Reduced pulmonary level of pathological vascular remodelling markers (α-actin, smoothelin-B) • Greater vascular density and luminal surface area of small pulmonary arteries • Reduced oxidative stress (nitrotyrosine) level in small pulmonary vessels
Gonzaléz-Candia et al. [55]	• Ewes subjected to chronic hypobaric hypoxia (altitude 3600 m) from pre-conception until end experiment • Tissue collected for analysis 29 d post-partum	• Oral to lamb: 1 mg/kg daily at 20:00 h from d 4–21 post-partum • Treatments: - Control (EtOH vehicle) - MEL	In MEL lambs compared with controls: • Lower pulmonary artery pressure for first 4 d of treatment. • Reduced contractile response to vasoconstrictors (K^+^, thromboxane synthase, endothelin) • Enhanced endothelium-dependent and muscle-dependent vasodilation in small pulmonary arteries • Reduced pulmonary oxidative stress markers (4-hydroxynonenal and nitrotyrosine)
Aridas et al. [56]	• Acute hypoxia: UCO at birth until blood pressure reached 18–20 mmHg • Brain magnetic resonance spectroscopy at 12 and 72 h post-partum • Lambs euthanased 72 h post-partum and tissue collected for analysis	• IV to lamb: 5 mg bolus 30 min post-partum + 5 mg every 2 h for 24 h (total 60 mg), or • 5 mg transdermal patch to lamb: 6 patches 30 min post-partum + 6 patches 12 h post-partum (total 60 mg) • Treatments: - Control (untreated) - Control + MEL - UCO - UCO + MEL (IV) - UCO + MEL-P (patch)	In UCO + MEL (IV) and UCO + MEL-P lambs compared with UCO: • Prevented 2.5–3-fold increase to magnetic resonance spectroscopy lactate: N-acetyl aspartate ratio • Attenuated grey and white matter apoptotic cell death, lipid peroxidation, and neuroinflammation • Lower latency to stand and suckle after birth, greater proportion of lambs standing and suckling, and reduced prevalence of seizures in asphyxiated lambs

*EtOH* Ethanol, *MEL* Melatonin, *NO* Nitric oxide, *UCO* Umbilical cord occlusion, *IV* Intravenous

#### Melatonin ameliorates IUGR in placental insufficiency and chronic hypoxia

The potential for maternal melatonin supplementation to ameliorate the effects of IUGR has been considered in intensive studies, over the past 5 years, in ovine models of chronic placental insufficiency and hypoxia (Table 2). Single umbilical artery ligation (SUAL), performed between gD105 and gD110 in sheep, induces chronic placental insufficiency and subsequent IUGR. Tare et al. [59] confirmed that SUAL induced growth restriction (bodyweight 75% of control fetuses after 7 d), along with significant reduction in fetal O_2_ saturation and PO_2_ in twin fetuses. Maternal melatonin infusion (2 mg bolus + 2 mg/h commencing 5–7 d after surgery) mitigated growth restriction of IUGR fetuses to 93% of control, and entirely normalised O_2_ saturation and PO_2_. Melatonin also prevented increases to ischaemia-reperfusion-induced infarct area seen in hearts of IUGR lambs, as well as enhancing ventricular contraction and coronary flow. In a second study using this model, where pregnancies proceeded until term, a lower dose of melatonin (6 mg/d) also normalised fetal O_2_ saturation and PO_2_, increased nitric oxide (NO) availability in coronary arteries, induced indomethacin-sensitive vasodilation, and prevented stiffening of coronary arteries in singleton IUGR lambs [59]. Other studies in this model found that infusing ewes with melatonin (6 mg/d from surgery until term) prevented chronic fetal hypoxia in IUGR lambs (birthweight 3.08 ± 0.47 vs. controls 4.51 ± 0.24 kg) [60], and protected cerebral perivascular cells in IUGR lambs (3.47 ± 0.36 vs. 4.37 ± 0.21 kg), thereby preventing blood-brain barrier disruption [61]. This was further evidenced in IUGR lambs (3.35 ± 0.34 vs. 4.49 ± 0.19 kg), where brains analysed 24 h post-partum exhibited cellular and axonal lipid peroxidation, white matter hypomyelination, and axonal damage; all of which were absent in IUGR lambs born to melatonin-treated ewes [43]. Further, while IUGR lambs took longer than controls to locate the udder and suckle after birth, these intervals were shortened significantly in the IUGR + melatonin treatment group [43]. This outcome in particular provides solid evidence that melatonin treatment provides the functional neurological improvement critical for maximising chances of lamb survival on farm.

**Table 2:** Experimental studies of prenatal melatonin supplementation in ovine models of IUGR and chronic hypoxia

Publication	Methodology	Melatonin dose rate/timing	Fetal/neonatal outcomes
Tare et al. (Study 1) [59]	• Induced IUGR (twins): SUAL at gD105–110 in one fetus only (other twin fetus constituted control) • Fetal femoral artery catheterised • 7 d after SUAL: Fetuses removed by C-section and heart function assessed *ex vivo* (Langendorff apparatus)	• IV to ewe: 2 mg bolus dose at 5 d after SUAL + 2 mg/h (48 mg/d) until post-mortem • Treatments: - Control (sham SUAL) - Control (sham SUAL) + MEL - SUAL - SUAL + MEL	In SUAL + MEL lambs compared with SUAL: • Mitigated growth restriction (93% of control weight vs. 75% of control weight) • Normalised fetal arterial O_2_ saturation and PO_2_ • Significantly increased basal coronary flow vs. control • Prevented 3-fold increase to ischaemia-reperfusion-induced infarct area in heart • Enhanced contractile function in right ventricle
Tare et al. (Study 2) [59]	• Induced IUGR (singletons): SUAL at gD105–110 • Fetal femoral artery catheterised and blood samples taken from 5 d after SUAL to gD140 • Ewes allowed to deliver naturally at term • Lamb hearts collected for analysis 24 h post-partum	• IV to ewe: 0.25 mg/h (6 mg/d) continuously from SUAL until term • Treatments: - Control (untreated) - Control + MEL - SUAL - SUAL + MEL	In SUAL + MEL lambs compared with SUAL: • Normalised fetal arterial O_2_ saturation and PO_2_ • Normalised NO bioavailability in coronary arteries • Induced indomethacin-sensitive vasodilation in coronary arteries • Prevented stiffening of coronary arteries
Polglase et al. [60]	• Induced IUGR: SUAL at gD105 • Fetal femoral artery catheterised and blood samples taken from 5 d after SUAL to gD145 • Ewes delivered naturally at term • Lamb lungs collected for analysis 24 h post-partum	• IV to ewe: 1 mg bolus 4 h after surgery + continuous infusion until term (6 mg/d) • Treatments: - Control (sham SUAL) - SUAL - SUAL + MEL	In SUAL + MEL lambs compared with controls and SUAL: • Reduced birthweight (3.17 ± 0.20 kg) vs. control (4.51 ± 0.24 kg) but not SUAL (3.08 ± 0.47 kg) • Prevented chronic hypoxia (similar to controls) • Similarly compromised lung structure (reduced secondary septal crest density and altered elastin deposition) as SUAL vs. control
Castillo-Melendez et al. [61]	• Induced IUGR: SUAL at gD105 • Ewes delivered naturally at term • Lamb brains collected for analysis 24 h post-partum	• IV to ewe: 1 mg bolus 4 h after SUAL + continuous infusion (6 mg/d) until term • Treatments: - Control (sham SUAL) - Control (sham SUAL) + MEL - SUAL - SUAL + MEL	In SUAL + MEL lambs compared with controls and SUAL: • Fewer laminin-positive blood vessel in subcortical and periventricular white matter vs. controls • Greater endothelium glucose transporter-1 immunoreactivity vs. SUAL (similar to controls) • Normalised pericyte coverage and astrocyte attachment to blood vessels vs. SUAL • Normalised apoptotic blood vessel number vs. SUAL • Prevented disruption of blood-brain barrier
Miller et al. [43]	• Induced IUGR: SUAL at gD105–110 • Fetal femoral artery catheterised and blood samples taken from 5 d after SUAL to gD145 • Ewes allowed to deliver naturally • Lambs brains collected for analysis 24 h post-partum	• IV to ewe: 1 mg bolus dose 4 h after surgery + IV infusion of 0.25 mg/h (6 mg/d) until delivery • Treatments: - Control (sham SUAL) - SUAL - SUAL + MEL	In SUAL + MEL lambs compared with SUAL: • Lower latency to stand and suckle after birth • Ameliorated oxidative stress, normalised myelination, and rescued axonopathy in brain
González-Candia et al. [62]	• Ewes subjected to chronic hypobaric hypoxia (altitude 3600 m) from pre-conception until end experiment • Ewes allowed to deliver naturally at term	• Oral to ewe: 10 mg/kg daily at 18:00 h from gD100 until term • Treatments: - Untreated IUGR (EtOH vehicle) - IUGR + MEL	In IUGR + MEL lambs compared with untreated IUGR: • Lower birthweight (2.88 ± 0.22 kg vs. 3.56 ± 0.16 kg) • Smaller biparietal diameter, crown-rump length, and abdominal diameter at birth
Gonzalez-Candia et al. [63]	• Ewes subjected to chronic hypobaric hypoxia (altitude 3600 m) from pre-conception until end experiment • Ewes delivered naturally at term • Lamb’s pulmonary artery catheterised 2 d post-partum • Lamb lungs collected for analysis at 12 d post-partum	• Oral to ewe: 10 mg/d from gD100 until term • Treatments: - Control (EtOH vehicle) - MEL	In MEL lambs compared with controls: • Lower birthweight (3.45 ± 0.36 kg vs. 4.90 ± 0.44 kg) • Lower biparietal diameter at birth, but recovered by 7 d post-partum • Higher plasma antioxidant capacity and lower pulmonary antioxidant activity • Lower ROS generation in lungs

*IUGR* intrauterine growth restriction, *SUAL* Single umbilical artery ligation, *gD* gestational day, *IV* Intravenous, *MEL* Melatonin, *NO* Nitric oxide, *EtOH* Ethanol, *ROS* Reactive oxygen species

Fetal growth is also restricted in ewes maintained under conditions of chronic hypobaric hypoxia equivalent to those at high altitude. Feeding melatonin (10 mg/kg daily) to ewes subjected to this chronic hypoxia from gD100 to term improved maternal antioxidant capacity and extended gestation length (155 ± 1 vs. 149 ± 1 d); however, this was accompanied by an unexpected and significant reduction in lamb birthweight (2.88 ± 0.22 vs. 3.56 ± 0.16 kg), biparietal diameter, crown-rump length, and abdominal diameter [62]. It is important to note the melatonin dose in this study, resulting in a 60 kg ewe receiving 600 mg melatonin daily, was ~ 100 times higher than the infusion dose given in most chronic hypoxia trials. The significant extension of gestation length in this study suggests that these very high melatonin doses may help delay the onset of parturition and prevent premature birth via inhibition of adrenocorticotropic hormone-induced cortisol release from the fetal adrenal gland [64, 65]. A lower dose of melatonin (10 mg/d, gD100 to term) in the same model increased plasma antioxidant capacity and decreased ROS production in the neonate, resulting in lower pulmonary antioxidant activity compared with lambs from untreated hypoxic ewes [63]. However, this was also accompanied by reduced birthweight (3.45 ± 0.36 vs. 4.90 ± 0.44 kg) and biparietal diameter. In both studies the authors were unable to explain melatonin-induced fetal growth restriction, but speculated it may involve impaired expression of nitric oxide synthase (NOS) and insulin-like growth factor 2 specific to high altitude sheep [62, 63]. Reduced fetal growth in melatonin-treated hypoxic ewes is in direct contrast to the melatonin-induced mitigation of fetal growth restriction in the SUAL model of IUGR [59], and normalised fetal biparietal distance and kidney size in the restrict-fed model of IUGR [66]. Melatonin-induced growth restriction has not been reported in any other ovine studies. Indeed, feeding 12 mg/d melatonin to ewes subject to constant light-induced suppression of endogenous melatonin release actually increased lamb birthweight (4.30 ± 0.18 vs. 3.68 ± 0.33 kg) [67]. Similarly, slow-release melatonin implants (18–36 mg at gD100) also increased fetal lamb weight at gD140 [68, 69], and in a rat model, melatonin (5 μg/mL drinking water) significantly improved birthweight of IUGR pups [70]. Overall these results suggest that melatonin promotes fetal growth even when the fetus is hypoxic, and only impairs fetal growth under conditions of chronic maternal hypoxia, but requires further studies to establish the underlying mechanisms in these conditions.

#### Maternal melatonin supplementation increases uterine blood flow

The ameliorative effects of melatonin on fetal growth restriction have been primarily linked to preservation of fetal oxygenation via enhanced uterine blood flow. However, despite several publications reporting melatonin-induced vasodilation, the underlying mechanisms have not been well understood until recently. The most commonly proposed mechanism involves the pleiotropic signalling molecule NO, which promotes vasodilation via relaxation of vascular smooth muscle [71]. NO bioavailability is reduced by ROS, specifically O_2_•^−^, which both inhibits endothelial NOS and removes NO by reacting with it to form the oxidant molecule peroxynitrite (ONOO^−^) [72]. Scavenging of ROS by melatonin and its metabolites therefore increases NO availability to promote vasodilation [71, 73–75], and increases sensitivity to vasorelaxants including bradykinin [76] and indomethacin [59]. An additional vasodilatory pathway for melatonin has recently been identified. As well as acting via increased NO availability, melatonin activates Ca^2+^-activated K^+^ (BK_Ca_) channels on smooth muscle endothelium both directly, via passage through cell membranes, and indirectly, via MT1 and MT2 receptors, to further promote vasorelaxation [77, 78]. These effects have been confirmed experimentally via Doppler ultrasonography. Melatonin fed to pregnant ewes (5 mg/d) increased umbilical artery blood flow at gD130 by 17% compared with non-treated ewes [66] and improved fetal uptake of branched chain amino acids involved in cell growth and protein synthesis [79]. Similarly, chronic uterine infusion of melatonin (~ 67 μg/d) from gD60 to gD90 in sheep increased both umbilical artery and fetal descending aorta bloodflow at gD90, and increased placental efficiency (fetal: placentome weight ratio) [80]. Melatonin may also influence fetal growth in late gestation by altering caruncle vascularity and RNA concentrations [81], as well as ratio of placentome types, with studies ongoing to elucidate these mechanisms [69].

#### Melatonin protects the fetus against acute hypoxia

In addition to minimising impacts of chronic hypoxia, melatonin also protects the fetal brain from acute hypoxia, which was deliberately induced via short periods of UCO (10–30 min) at various points throughout late gestation across multiple studies (Table 3). Ten min of UCO at gD124–127 triggered a rapid increase of •OH release in fetal grey matter for 60–90 min after occlusion, with an additional spike 6–8 h later lasting 2–3 h [45]; a biphasic pattern characteristic of HIE [14, 15]. Melatonin supplied intravenously to ewes (1 mg bolus 1 h before UCO + 1 mg/h for 2 h) abrogated •OH release and reduced the amount of lipid peroxidation in various regions of the fetal brain [45]. Intravenous maternal administration of melatonin similarly reduced fetal brain lipid peroxidation in other UCO trials, as well as preserving blood-brain barrier integrity, reducing microglial activation and astrogliosis, and increasing oligodendrocyte number [82, 83]. Similar outcomes were also observed when melatonin was infused directly to the fetus following UCO, including lower lipid peroxidation, microglial activation, and apoptosis [84, 85]. This was accompanied by region-specific increases in oligodendrocyte cell number and myelin density in fetal white matter, and neuronal survival in the cortex [85]. These studies provide strong evidence of melatonin’s neuroprotective effects in fetuses exposed to acute hypoxia in utero, even with brief treatment periods (2–6 h), but effects of melatonin on lambs subjected to intra-partum hypoxia have not yet been tested. Given what is known, including potent neuroprotection even during total UCO and oxygen deprivation, it is reasonable to hypothesise that melatonin treatment could provide similar benefit to neonates exposed to the conditions of prolonged parturition.

**Table 3:** Experimental studies of prenatal melatonin supplementation in ovine models of acute hypoxia

Publication	Methodology	Melatonin dose rate/timing	Fetal/lamb outcomes
Miller et al. [45]	• Induced fetal asphyxia: 10 min UCO at gD124–127 • Probes fitted to fetal brains • Fetal brains collected for analysis 48 h after UCO	• IV to ewe: 1 mg bolus + 1 mg/h for 2 h only • Treatments: - Sham + vehicle (EtOH) - UCO + vehicle - UCO + MEL	In UCO + MEL fetuses compared with UCO + vehicle: • Prevention of 60–90 min •OH spikes observed in grey matter of UCO + vehicle fetuses immediately after UCO and 8–9.5 h after UCO • Significantly reduced lipid peroxidation in cortical white matter, thalamus/hypothalamus, and subcallosal bundle
Drury et al. [82]	• Induced fetal asphyxia: 25 min UCO at gD101–104 • Fetal brains collected for analysis 7 d after UCO	• IV to ewe: 0.1 mg/kg bolus 15 min before UCO + 0.1 mg/kg/h for 6 h • Treatments: - Sham UCO - UCO + saline - UCO + vehicle (EtOH) - UCO + MEL	In UCO + MEL fetuses compared with UCO + saline and UCO + vehicle: • Significantly faster electroencephalogram recovery vs. UCO + saline • Delayed onset of seizures • Greater number of mature oligodendrocytes (similar level as sham UCO) • Reduced microglial activation in white matter
Yawno et al. [83]	• Induced fetal asphyxia: 10 min UCO at ~gD130 • Fetal brains collected for analysis 48 h after UCO	• IV to ewe: 1 mg bolus 1 h before UCO + 2 mg/h for 2 h (total 5 mg) • Treatments: - Sham UCO + vehicle (EtOH) - UCO + vehicle (EtOH) - UCO + MEL	In UCO + MEL fetuses compared with UCO + vehicle: • Ameliorated pyknotic cell death in hippocampus (> 7-fold) and cerebellum (3-fold) • Prevention of astrogliosis, albumin uptake increases, microglial activation and lipid peroxidation in brain
Welin et al. [84]	• Induced fetal asphyxia: 23.5 min UCO at gD91–93 • Fetal brains collected for analysis 4 d after UCO	• IV to fetus: 20 mg/kg hourly from 10 min after UCO for 6 h • Treatments: - UCO + EtOH vehicle - UCO + MEL	In UCO + MEL fetuses compared with UCO + EtOH vehicle: • Attenuated increase of oxidative stress (8-isoprostane) in serum • Fewer apoptotic (TUNEL-positive) cells in subcortical white matter and thalamus • Fewer activated microglial cells in white matter
Yawno et al. [85]	• Induced fetal asphyxia: 25 min UCO at gD102 • Fetal femoral artery + vein catheterised • Fetal brains collected for analysis 10 d after UCO	• IV to fetus: 0.2 mg bolus 2 h after UCO + 0.1 mg/h for next 24 h (total 2.6 mg) • Treatments: - Control (Sham UCO + saline) - UCO + saline - UCO + MEL	In UCO + MEL fetuses compared with UCO + saline: • Prevented increase to white matter cell death • Reduced microglial activation (neuroinflammation) in subventricular and subcortical white matter • Prevented increase of oxidative stress (8-OHdG+) in subventricular and subcortical white matter • Normalised oligodendrocyte number in periventricular white matter • Normalised CNPase+ myelin density in subcortical white matter

*UCO* Umbilical cord occlusion, *gD* gestational day, *IV* Intravenous, *EtOH* Ethanol, *MEL* Melatonin, *TUNEL* Terminal deoxynucleotidyl transferase dUTP nick end labelling, *8-OHdG* 8-hydroxy-2′-deoxyguanosine

#### Melatonin enhances BAT accumulation and function

BAT activation at birth is triggered by norepinephrine released in response to cold exposure [86]. Melatonin directly inhibits the response of BAT to norepinephrine in utero, thereby promoting BAT accumulation via prevention of premature lipolysis [64], and several intervention studies have therefore assessed effects of manipulating maternal melatonin on BAT accumulation and thermogenesis in the lambs (Table 4). Interestingly, Seron-Ferre et al. [67] demonstrated that experimental melatonin suppression throughout gestation, via a constant lighting schedule, reduced neonatal BAT reserves by around 50% compared with lambs gestated by ewes housed under a 12 h light:12 h dark schedule. Additionally, BAT from lambs born to ewes on the constant lighting schedule had higher basal rates of lipolysis and was non-responsive to norepinephrine. Importantly, lambs gestated by ewes exposed to constant lighting but receiving a daily melatonin supplement (12 mg at 17:00 h) exhibited no impairment to BAT accumulation or function during *in vivo* cold challenge or *ex vivo* tissue analysis (both 4–6 d post-partum), further reinforcing the importance of melatonin for BAT development. Further, lambs born to melatonin-treated ewes were 11–15% heavier than lambs from untreated ewes exposed to the split or constant lighting schedules, respectively [67]. The sensitivity of BAT to metabolic challenges appears dependent on melatonin’s regulation of key adipogenic and thermogenic genes, such that neonatal BAT function is impaired by the absence of melatonin during gestation, and restored, or even enhanced, when melatonin is reintroduced [67, 87, 88]. The relationship between melatonin and BAT accumulation was also demonstrated by Sales et al. [68], who reported that implanting ewes with a slow release melatonin implant (Regulin®, 18 mg) at gD100 increased fetal BAT stores at gD140 by 18% and 35% for singletons and twins, respectively. This was accompanied by similar increases in fetal weight (Table 4) [68, 69]. These outcomes suggest melatonin may enhance thermogenic capacity of the neonatal lamb, which is critical for survival in colder environments.

**Table 4:** Experimental studies of melatonin effects on fetal growth and brown adipose tissue accumulation/function

Publication	Methodology	Melatonin dose rate/timing	Fetal/lamb outcomes
Seron-Ferre et al. [67]	• Daily lighting schedule manipulation from ~gD93 until term • Lambs instrumented 2 d post-partum • 4–6 d post-partum: lambs exposed to 24 °C for 1 h, 4 °C for 1 h, 24 °C for 1 h before BAT collected for analysis	• Oral to ewe: 12 mg daily at 17:00 h from ~gD93 until term • Treatments: - LD: 12 h light + 12 h dark - LL: 24 h constant light - LL + MEL: 24 h constant light + melatonin	In LL + MEL lambs compared with LL: • Higher birthweight (4.30 ± 0.18 kg) vs. LL (3.68 ± 0.33 kg) and LD (3.85 ± 0.27 kg) • Normalised BAT and skin temperature at thermoneutral (24 °C) and cold (4 °C) ambient temperatures (similar to control) • Mitigated increase to norepinephrine level during cold exposure (4 °C) • Normalised perirenal BAT weight (similar to control) • *Ex vivo*: Normalised bat function (LL exhibited increased basal lipolysis, no response to norepinephrine, and increased expression of adipogenic/thermogenic and clock genes)
Sales et al. [68]	• Commercial grazing conditions on natural pasture • Fetal analysis at gD140	• Subcutaneous to ewe: 18 mg per slow-release implant (Regulin®) at gD100 • Treatments: - MEL0: control - MEL1: one implant (18 mg) - MEL2: two implants (36 mg)	In fetuses: • Singletons: BAT weight 18% higher in MEL2 fetuses vs. both MEL0 and MEL1 • Twins: BAT weight 35% higher in MEL1 fetuses vs. both MEL0 and MEL2 • All litter sizes: MEL2 fetuses 5–8% heavier with larger thorax diameter and crown-rump length vs. both MEL0 and MEL1
Sales et al. [69]	• Commercial grazing conditions on natural pasture • Fetal analysis at gD140	• Subcutaneous to ewe: 18 mg per slow-release implant (Regulin®) at gD100 • Treatments: - M0: control - M1: one implant (18 mg) - M2: two implants (36 mg)	In fetuses: • MEL2 male twins 14% or 22% heavier than MEL0 and MEL1, respectively • All litter sizes: MEL2 umbilical cord PO_2_ 18–20% higher vs. both M0 and M1

*gD* gestational day, *BAT* Brown adipose tissue, *MEL* Melatonin

## Conclusion

Pre-weaning mortality remains high globally at 15–20% of all lambs born with there being little to no improvement over the past 40 years. As well as the significant economic impacts of lost production, this also constitutes a major welfare issue. As such, there is a clear requirement for the development of novel and effective strategies to reduce neonatal loss to improve welfare, production, and income. Melatonin supplementation during pregnancy minimises the risk of IUGR and neurological abnormalities during fetal development via improved uterine blood flow and potent antioxidant effects. Additionally, melatonin protects the fetal brain from acute hypoxia, which is likely to be of benefit during prolonged parturition. Further, melatonin has the potential to improve thermoregulation of the neonatal lamb by increasing BAT reserves at birth. The existing literature provides strong evidence of a role for melatonin in improving neurological outcomes for lambs that experience a compromised gestation and/or prolonged parturition, though to date there has been very little investigation into how this translates to livestock production. As such, there is a clear basis for continued research into the effects of maternal melatonin supplementation during gestation on physiological parameters associated with lamb survival after birth, specifically birthweight, early suckling behaviour, and thermoregulation, and determining the optimal delivery method to achieve this. While positive results have been reported in studies using intravenous infusion, oral delivery, and slow-release implants, practicality must be a major consideration for livestock production systems. Therefore, oral delivery and slow-release implants appear the most attractive options for future research, with potential for subsequent investigation into pre-weaning survival under commercial production conditions. The other main areas of interest include determining how effects vary with differences in litter size, ewe condition, lambing season, and breed.

## Data Availability

Not applicable.

## Abbreviations

ATP: Adenosine triphosphate
BAT: Brown adipose tissue
gD: gestational day
HIE: Hypoxic-ischaemic encephalopathy
IUGR: Intrauterine growth restriction
NO: Nitric oxide
NOS: Nitric oxide synthase
O_2_•^−^: Superoxide
•OH: Hydroxyl
ROS: Reactive oxygen species
SUAL: Single umbilical artery ligation
UCO: Umbilical cord occlusion
UCP1: Uncoupling protein 1
